# Genomic evolutional analysis of surgical resected specimen to assess osimertinib as a first‐line therapy in two patients with lung cancer harboring *EGFR* mutation: Case series

**DOI:** 10.1111/1759-7714.15241

**Published:** 2024-02-07

**Authors:** Hayato Koba, Taro Yoneda, Hiroko Morita, Hideharu Kimura, Yuya Murase, Nanao Terada, Yuichi Tambo, Masafumi Horie, Kazuo Kasahara, Isao Matsumoto, Seiji Yano

**Affiliations:** ^1^ Department of Respiratory Medicine Kanazawa University Hospital Kanazawa Japan; ^2^ Respiratory Medicine, Komatsu Municipal Hospital Komatsu Japan; ^3^ Respiratory and Allergic Medicine, Morita Hospital Komatsu Japan; ^4^ Department of Molecular and Cellular Pathology Graduate school of Medical Sciences, Kanazawa University Kanazawa Japan; ^5^ Department of Pulmonary Medicine and Oncology Graduate School of Medicine, Nippon Medical School Tokyo Japan; ^6^ Department of Thoracic Surgery Kanazawa University Kanazawa Japan

**Keywords:** AXL, epidermal growth factor receptor mutation, genomic evolution, intrinsic resistance, osimertinib

## Abstract

Epidermal growth factor receptor (EGFR) tyrosine kinase inhibitor (TKI) is crucial for patients with lung cancer harboring *EGFR* mutations. However, almost all patients experience disease progression, regardless of their response to the targeted therapy, necessitating the development of additional treatment options. Two patients with lung cancer harboring *EGFR*‐L858R mutations in exon 21 were treated by surgical resection during successful osimertinib treatment. Because the pathological diagnosis was suspected to be pleural metastasis, osimertinib treatment was continued until disease progression. We analyzed the evolution of genomic alterations and the levels of AXL using tumor specimens obtained by repeated biopsies during the course of treatment: initial diagnosis, operation, and disease progression. Genetic alterations detected at the three time points were dramatically changed and showed reductions in numbers, while *EGFR*‐L858R mutations were detected in all samples tested in both patients. Immunohistochemical expression of AXL remained positive from the beginning of analysis to disease progression. Clonal evolution under oncogenesis is related to gradual accumulation of genomic alterations during tumor growth. However, our case series revealed that volume reduction procedures may cause this phenomenon. Therefore, identification of intrinsic drug‐resistant cells in tumors may be as important as detection of acquired genetic alterations.

## Graphical abstract

Gene aberrations alter in form dramatically; clonal evolution Clinical presentation of intrinsic resistant mechanism.

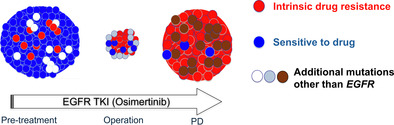

## INTRODUCTION

The third‐generation epidermal growth factor receptor (EGFR) tyrosine kinase inhibitor (TKI) osimertinib is a standard treatment for metastatic lung cancer harboring *EGFR* mutations, such as exon 19 deletion and L858R, according to the FLAURA study.^1^ Although osimertinib therapy does provide a survival benefit to these patients, complete response rates are still very low. Furthermore, the ADAURA study recently reported that adjuvant osimertinib therapy to patients with completely resected lung cancer harboring *EGFR* mutation provided a significant overall survival benefit.^2^ Further innovative treatments, such as multimodal therapy, are needed.

In this study, we describe the treatment course and genomic analysis of two patients with the *EGFR*–L858R mutation who underwent surgical resection during osimertinib treatment.

## CASE REPORT

**FIGURE 1:**
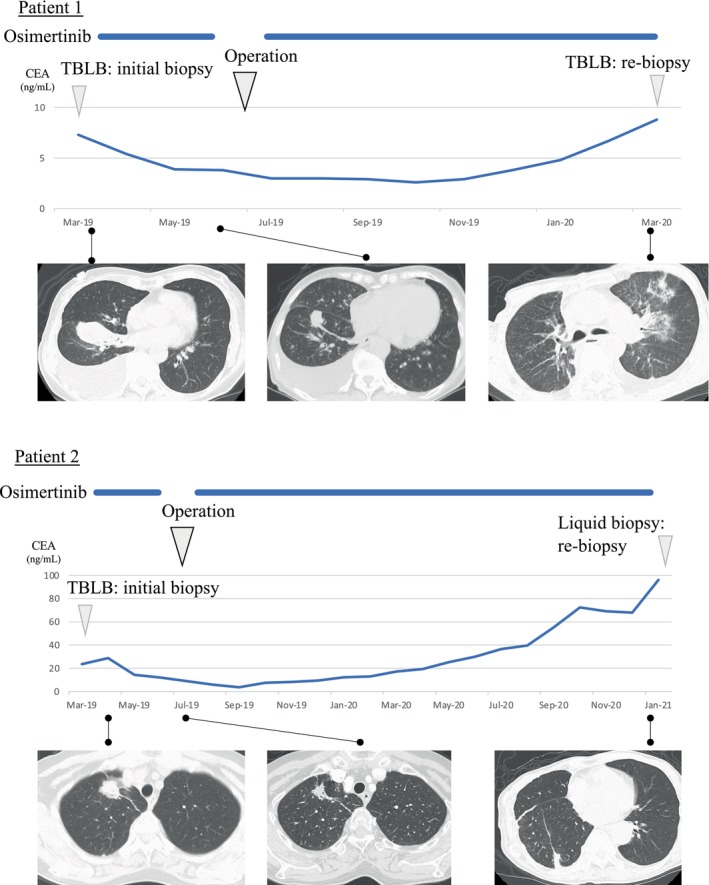
Clinical course. Both patients continued osimertinib treatment until disease progression, with a few months perioperative cessation. TBLB, transbronchial lung biopsy.

Patient 1 (a 73‐year‐old woman) and patient 2 (a 74‐year‐old woman) were both diagnosed with primary lung cancer harboring the *EGFR* mutation L858R in exon 21. The patients were diagnosed with cT2aN3M1a PLE stage lVA and cT4N2M1a PLE stage lVA disease, respectively. Initially, patients received osimertinib as a first‐line therapy because their tumors were considered to be unresectable based on staging, although cytologies of pleural effusion were negative (Figure 1). Subsequently, tumor shrinkage was observed in both patients, and the treatment plan was reconsidered through a multidisciplinary discussion. There were no extrathoracic metastatic lesions or pleural nodules assessed as malignant metastases by computed tomography scanning, suggesting negative findings for malignancy; therefore, surgery was concluded to be an option for both patients. This information was explained, and removal of the remaining tumor lesion was proposed as a treatment option. Finally, lobectomy with pleurectomy was performed for both patients after 4 months of osimertinib therapy after obtaining informed consent for the operation. Unfortunately, for both patients, surgical and postoperative diagnoses revealed positivity for pleural dissemination, and the pathological stage was diagnosed as stage lVA. Subsequently, osimertinib was administered again as a systemic chemotherapy beginning a few weeks after the surgical procedure in both patients. Four months later, left lung infiltration was observed in patient 1, and disease progression was detected via transbronchial‐lung biopsy. In the case of patient 2, pleural thickening and pleuralgia appeared 14 months later. Although tissue biopsy was unachievable, we estimated disease progression clinically and obtained plasma samples as liquid biopsies at that time.

To further investigate the molecular transition from initial diagnosis to recurrence through surgical resection, target genotyping assays using next‐generation sequencing and evaluation of relevant protein expression were performed. The protocol for the analysis was approved by the Institutional Review Board of the Human Genome/Gene Analysis Research Ethics Committee of Kanazawa University (approval no. 434) and was in compliance with the guidelines established in the Declaration of Helsinki. Both patients provided written informed consent for participation in the study. Tumor DNA samples were extracted from both patients at three phases: initial diagnostic biopsy, surgical resection, and biopsy at disease progression. DNA samples from normal lung tissues at operation were exploited to detect germline mutations. The details of reagents and equipment used for sequencing and immunohistochemical (IHC) staining are shown in Tables S1 and S2.

**FIGURE 2:**
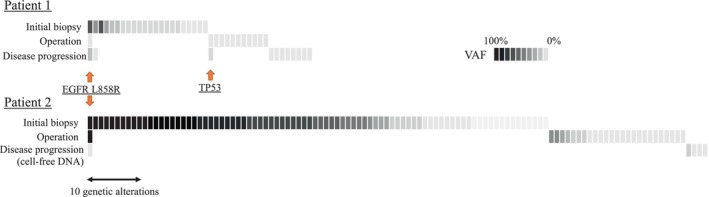
Heat map of genetic alterations. Bars represent genomic alterations, and depth indicates VAF. VAF, variant allele frequency.

Mutations detected in normal lung tissues (i.e., germline mutations) were eliminated, leaving 22, 12, and 10 somatic mutations detected from biopsies in patient 1 and 84, 26, and 5 somatic mutations detected from patient 2 (Figure 2). The unique mutation detected throughout all three phases was a sensitive *EGFR* mutation, L858R, in both cases. These new genetic alterations detected at disease progression were observed in both cases; however, another alteration in *TP53* was detected at surgical resection in patient 1. The mutation burden was reduced, and almost all original mutations were eliminated as the disease progressed in each case. The genetic alterations that were detected after acquisition of osimertinib resistance were observed in ataxia telangiectasia mutated (*ATM*), *KMT2D* (three types of mutations; a COMPASS‐like enzyme also called MLL4), *BAI3*, and *CDKN2A* in patient 1 and *AMER1* (two types of mutations) and *CREBBP* in patient 2 (Table 1).

**TABLE 1:** Additive genetic alterations detected after acquisition of osimertinib resistance.

Patient	Gene symbol	Chr	Position	Annotation	Transcript type	HGVS.c	HGVS.p
1	*BAI3*	6	69 942 494	Missense variant	Protein coding	c.71 T > C	p.Leu24Pro
	*CDKN2A*	9	21 974 675	Splice donor variant and intron variant	Protein coding	c.150 + 2 T > G	‐
	*ATM*	11	108 196 957	Upstream gene variant	Retained intron	n.‐1163delT	‐
	*KMT2D*	12	49 426 729	Disruptive in‐frame deletion	Protein coding	c.11756_11758delAGC	p.Gln3919del
	*KMT2D*	12	49 427 686	Frameshift variant	Protein coding	c.10801delC	p.Gln3601fs
	*KMT2D*	12	49 433 805	Missense variant	Protein coding	c.7748C > T	p.Ala2583Val
2	*CREBBP*	16	3 808 052	Splice region variant and intron variant	Protein coding	c.3370‐4delT	‐
	*AMER1*	X	63 410 181	Missense variant	Protein coding	c.2986A > C	p.Ile996Leu
	*AMER1*	X	63 411 180	Missense variant	Protein coding	c.1987G > C	p.Val663Leu

Abbreviation: Chr, chromosome.

**FIGURE 3:**
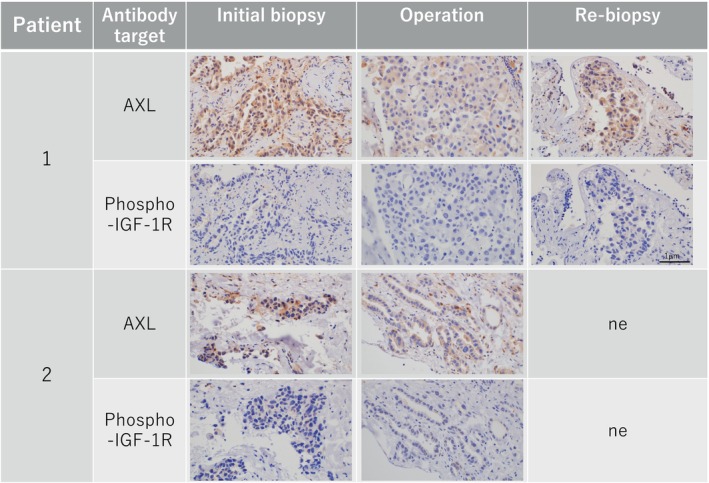
Immunohistochemical staining results. AXL expression was consistently positive from pretreatment to operation and disease progression. All phospho‐IGF‐1R expression was negative. ne, not examined; phospho‐IGF‐1R, phosphorylated insulin‐like growth factor‐1 receptor.

Tumor specimens for IHC were available at all three points in patient 1, while the specimens at the initial biopsy and operation were available in patient 2. We detected weak AXL expression in tumor cells at initial biopsy in both patients (Figure 3). Of interest, we observed discernibly higher AXL expression in tumor cells at operation in both patients, which is highly consistent with our previous reports showing that AXL plays crucial role for emerging osimertinib tolerant cells.^3^, ^4^ On the other hand, we did not detect phosphorylated insulin‐like growth factor‐1 receptor (phospho‐IGF‐1R) expression the specimens in patient 1 or 2.

## DISCUSSION

Here, we present two rare cases of metastatic lung cancer harboring *EGFR* mutations in patients who underwent surgical resection during successful osimertinib treatment. Unintentionally, initial osimertinib therapies to both patients were dealt neoadjuvant chemotherapy. There was fortunately no serious adverse events before and after operations, but further investigation is needed to clarify the efficacy and safety of neoadjuvant osimertinib therapy.^5^ We inferred that pleurectomy could bring no recurrence of pleural effusion after surgery. Although pleurectomy toward malignant pleural effusion is controversial, several reports have described the efficacy of the technique.^6^, ^7^ Not only pleurectomy but also targeted therapy: osimertinib might therefore provide a good control for our patients.

In both patients, the original sensitive L858R mutation in *EGFR* was the only consistent mutation detected throughout the osimertinib treatment course. Notably, the number of alterations was reduced during the treatment course. The resistance mechanisms to first‐line osimertinib therapy have previously been studied in detail.^8^ ATM has been shown to play essential roles in sensing DNA double‐stranded breaks and coordinating their repair,^9^ whereas KMT2D is a histone lysine methyltransferase whose loss of function promotes lung tumorigenesis in mice.^10^ BAI3 protein is structurally altered and a potential therapeutic target in small cell lung cancer.^11^ *CDKN2A* aberrations are another osimertinib resistance mechanism and are a negative predictor of response to immunotherapy.^8^, ^12^ Additionally, *AMER1* has been reported to function as a tumor‐suppressor gene in Wilms' tumor,^13^ and loss of AMER1 regulates colorectal cancer progression and metastasis.^14^ Loss of *CREBBP* has been shown to reduce histone acetylation and transcription of cellular adhesion genes in small cell lung cancer, whereas MUC16, which is related to many types of cancers, increases metastasis via modulation of E‐cadherin, N‐cadherin, and vimentin expression.^15^ Out of alterations presented in Table 1, *CDKN2A* aberrations are known as one of them and a negative predictor of response to immunotherapy,^8^, ^12^ whereas gene aberrations of ATM, KMT2D, BAI3, AMER1 and CREBBP have not been previously reported as far as we know. These acquired genetic alterations may be associated with the osimertinib resistant mechanism.

We hypothesized that gene alterations detected from shrunken tumors may be crucial for tumor proliferation owing to acquired resistance to molecular‐targeted drugs. However, our findings showed that gene alterations detected during the time from initial diagnosis to operation and disease progression were dynamically different. Additionally, almost all novel mutations at surgical resection were abolished, and other gene aberrations were generated at disease progression (Figure 2). Clonal evolution under oncogenesis involves gradual accumulation of drug resistance‐related genomic alterations during tumor growth.^16^ However, our case series revealed contradictory findings. Thus, we suggest that volume reduction procedures may be related to this phenomenon. Another potential explanation is that genetic alterations originating from unresectable lesions, including pleural dissemination, were never detected, and the primary lesion was removed. Our case series showed that at the time of tumor shrinkage, it was difficult to predict the additional genetic aberrations related to disease recurrence. Drug tolerance is the basis for acquired resistance to EGFR‐TKIs including osimertinib. We recently reported that AXL and phospho‐IGF‐1R could play mutually exclusive roles on emergence of tolerant cells to osimertinib.^3^, ^4^ In the AXL high expressing *EGFR* mutated NSCLC cells, SPRY4 protein, a negative regulator of MAPK pathway, inhibits activation of AXL, while suppression of MAPK pathway by osimertinib adversely activates AXL by inhibiting expression of AXL, resulting in emergence of tolerant cells by restoring MAPK signaling.^3^, ^4^ In the present study, we observed expression of AXL but not phospho‐IGF‐1R in tumor specimens obtained at both initial biopsy as well as operation in two patients. Collectively, these findings strongly suggest that AXL may contribute to emergence of osimertinib‐tolerant tumor cells which were observed in surgical specimens obtained from two patients. The preclinical study reported that the combination of AXL inhibitor and EGFR‐TKIs could be highly potent when given as the initial treatment for AXL‐high expressing EGFR mutated NSCLC.^17^ Based on this preclinical evidence, a clinical trial to evaluate the safety and efficacy of the first‐line treatment with AXL inhibitor ONO7475‐03 and osimertinib in *EGFR* mutated NSCLC patients (jRCT2051210045) is currently ongoing.

There were some limitations to this study. First, it was a case series with only two cases. Second, the number of genes assessed in the lung cancer panel was small. Third, the biopsy specimens obtained from the patients, with the exception of the surgical resection specimens, were too small to assess the genetic features of the entire tumor.

In conclusion, we present two rare cases of metastatic lung cancer harboring *EGFR* mutations in patients who underwent surgical resection during successful osimertinib treatment safely. Our results show that it was difficult to predict additional genetic aberrations related to disease recurrence because of the high variation among the genetic alterations. Accordingly, identification of intrinsic drug‐resistance components at initial diagnosis may be more important for improving outcomes in patients with *EGFR* mutant lung cancer.

## AUTHOR CONTRIBUTIONS

Hayato Koba and Isao Matsumoto: Conception and design: Hayato Koba, Taro Yoneda, Hiroko Morita, Kazuo Kasahara, and Isao Matsumoto: Administrative support: of study materials or patients: Collection and assembly of data: Hayato Koba, Nanao Terada, and Masafumi Horie. Hayato Koba, Hideharu Kimura, Masafumi Horie, Kazuo Kasahara, and Seiji Yano: Data analysis and interpretation: All authors were responsible for writing the manuscript and its final approval.

## FUNDING INFORMATION

H. Ko thanks the Japan Society for the Promotion of Science KAKENHI Grant Number JP: 22K20788 for support. Y. T thanks the Japan Society for the Promotion of Science KAKENHI grant no. JP: 22K15577 for support.

## CONFLICT OF INTEREST STATEMENT

H. Ko reports the relevant financial activities outside the submitted work; personal fees from AstraZeneca K. K. Y. T reports the relevant financial activities outside the submitted work; personal fees from AstraZeneca K. K., Chugai Pharmaceutical, TAIHO Pharmaceutical, MSD, Bristol Myers Squibb. The other authors declare no conflict of interest.

## Supporting information


**Table S1.** Reagents and equipment.

**Table S2.** Gene list of targeted sequence.

## Data Availability

All of the data that support the findings of this study are available.
